# Draft genome sequence of *Bacillus velezensis* endophytically isolated from roots of *Polygala paniculata*

**DOI:** 10.1128/mra.00826-24

**Published:** 2024-09-16

**Authors:** Felipe de Paula Nogueira Cruz, Paulo Henrique Marques de Andrade, Cristina Paiva de Sousa, Paulo Teixeira Lacava

**Affiliations:** 1 Natural and Physical Sciences Department, Graceland University, Lamoni, Iowa, USA; 2 Laboratory of Microbiology and Biomolecules—LaMiB, Department of Morphology and Pathology, Federal University of São Carlos, São Carlos, São Paulo, Brazil; 3 Biotechnology Post Graduate Program, Federal University of São Carlos, São Carlos, São Paulo, Brazil; Department of Biology, Queens College, Queens, New York, USA

**Keywords:** *Polygala*, endophytes, Atlantic forest, medicinal plants

## Abstract

*Polygala paniculata* is a medicinal plant that harbors a remaining unknown microbiome. Those plants are known for their analgesic and anti-inflammatory properties and are widely found in the Brazilian Atlantic Forest. Herein, we report the isolation of the endophyte *Bacillus velezensis* GPP30 with a draft genome estimated at 4.0 Mpb.

## ANNOUNCEMENT

*Bacillus velezensis* is a versatile and skilled producer of bioactive compounds that can enhance plant growth and systemic resistance by phytopathogen antagonism (1–3). *B. velezensis* GPP30 was endophytically isolated from *Polygala paniculata* roots. Plants were collected in the city of Peruibe, south coast of São Paulo State, Brazil (Latitude: −24° 19′12″ S/ Longitude: 46° 59′ 54″ W) (SisGen—registration number: AF1A75A) (4). GPP30 was isolated after superficial disinfection and incubation of grounded plant structures in phosphate-buffered solution, which were serially diluted and inoculated on tryptic soy agar with benomyl (50 µg/mL) and incubated at 28°C until growth (5, 6). GPP30 genomic DNA was extracted using a PowerSoil Kit (Qiagen) after a lysis process of heat and freeze cycles at 65°C and −80°C for 15 minutes (4).

Libraries were prepared using the Nextera XT DNA Library Prep, following the manufacturer’s instructions, and tagmented with Nextera transposome at 55°C for 5 minutes, followed by PCR amplification with 12 cycles using Nextera XT Indexes. Library concentration and size were analyzed by Qubit dsDNA High Sensitivity and Bioanalyzer High Sensitivity DNA Kits (Agilent). Sequencing was performed on the NextSeq 550 (Illumina) using the NextSeq 500/550 Mid Output kit v2.5 with 150 cycles.

Quality analysis was made using FastQC—UniPro UGENE v. 34 (7); *de novo* assembly of 1,602,705 reads was made by SPAdes Genome Assembler v. 3.12.0 (8) applying kmers 21, 33, and 55 followed by quality assurance using QUAST Genome assembly Quality v. 5.0.2 (9). The final contig with a total size of 4,337.498 bp (N50 154942) and 45.53% of GC content was annotated using Rast v. 2.0 (10) and was completed by PGAP. A total of 4,440 predicted genes were distributed in 4,312 protein-coding genes, 3 S rRNA, 88 noncoding RNAs, 36 tRNAs, and 88 misc RNAs.

Comparative genomic analysis using the 16S rRNA, rpoB gene, and average nucleotide identity (11, 12) showed a strong identity of GPP30 with *B. velezensis*. In contrast, the tetra nucleotide frequency signature (http://jspecies.ribohost.com/jspeciesws/) (13–15) exhibited correlation coefficients of 0.99972 for *B. velezensis* and 0.99974 for *B. amyloliquefaciens*. Z-scores of the tetra correlation search were 0.99999 and 0.99974, which corroborates its 99% phylogenetic similarity with *B. subtilis* and *B. amyloliquefaciens*, respectively (16).

Additional analysis using PathogenFinder 1.1 (17) and ResFinder 2.1 (18) described its low probability of being a human pathogen by the absence of antibiotic resistance genes. AntiSMASH 7.0 (19) revealed the presence of coding regions for polyketides (surfactin and difficidin), nonribosomal peptides (bacillibactin and fengycin), terpenes, and bacilysin.

In summary, the potential of endophytes associated with *P. paniculata* remains unknown, and a better understanding of this niche is fundamental since such symbiont microbes can naturally enhance growth and protect the host plant against diseases by the production and biodisponibilization of nutrients (20).

## Data Availability

The genome sequence of *B. velezensis* GPP30 has been deposited in GenBank under the accession number JAUKPF000000000. The assembly and raw reads (SRR25630284) are collected under BioProject number PRJNA991089.

## AFTER EPUB

*[This article was published on 16 September 2024 with errors in the funding information. The author who received funding was corrected in the current version, posted on 10 October 2024.]*
